# Fc-Mediated Antibody Effector Functions During Respiratory Syncytial Virus Infection and Disease

**DOI:** 10.3389/fimmu.2019.00548

**Published:** 2019-03-22

**Authors:** Elisabeth A. van Erp, Willem Luytjes, Gerben Ferwerda, Puck B. van Kasteren

**Affiliations:** ^1^Centre for Infectious Disease Control, National Institute for Public Health and the Environment (RIVM), Bilthoven, Netherlands; ^2^Section Pediatric Infectious Diseases, Laboratory of Medical Immunology, Radboud Institute for Molecular Life Sciences, Nijmegen, Netherlands; ^3^Radboud Center for Infectious Diseases, Nijmegen, Netherlands

**Keywords:** RSV, antibody, Fc gamma receptor, Fc-mediated effector functions, antibody functionality, ADCC, ADCP, vaccine

## Abstract

Respiratory syncytial virus (RSV) is a major cause of severe lower respiratory tract infections and hospitalization in infants under 1 year of age and there is currently no market-approved vaccine available. For protection against infection, young children mainly depend on their innate immune system and maternal antibodies. Traditionally, antibody-mediated protection against viral infections is thought to be mediated by direct binding of antibodies to viral particles, resulting in virus neutralization. However, in the case of RSV, virus neutralization titers do not provide an adequate correlate of protection. The current lack of understanding of the mechanisms by which antibodies can protect against RSV infection and disease or, alternatively, contribute to disease severity, hampers the design of safe and effective vaccines against this virus. Importantly, neutralization is only one of many mechanisms by which antibodies can interfere with viral infection. Antibodies consist of two structural regions: a variable fragment (Fab) that mediates antigen binding and a constant fragment (Fc) that mediates downstream effector functions via its interaction with Fc-receptors on (innate) immune cells or with C1q, the recognition molecule of the complement system. The interaction with Fc-receptors can lead to killing of virus-infected cells through a variety of immune effector mechanisms, including antibody-dependent cell-mediated cytotoxicity (ADCC) and antibody-dependent cellular phagocytosis (ADCP). Antibody-mediated complement activation may lead to complement-dependent cytotoxicity (CDC). In addition, both Fc-receptor interactions and complement activation can exert a broad range of immunomodulatory functions. Recent studies have emphasized the importance of Fc-mediated antibody effector functions in both protection and pathogenesis for various infectious agents. In this review article, we aim to provide a comprehensive overview of the current knowledge on Fc-mediated antibody effector functions in the context of RSV infection, discuss their potential role in establishing the balance between protection and pathogenesis, and point out important gaps in our understanding of these processes. Furthermore, we elaborate on the regulation of these effector functions on both the cellular and humoral side. Finally, we discuss the implications of Fc-mediated antibody effector functions for the rational design of safe and effective vaccines and monoclonal antibody therapies against RSV.

## Introduction

Respiratory syncytial virus (RSV) infection is a major cause of severe respiratory illness requiring hospitalization in young infants (1). Hospitalization for severe RSV-mediated disease peaks between 6 weeks and 6 months of life (2, 3), when infants mainly depend on their innate immune system and maternal antibodies for protection against infectious diseases. However, the exact role of RSV-specific maternal antibodies is unclear. Some studies show that high titers of maternal antibodies are associated with protection against RSV infection (4–6); whereas others indicate that high maternal antibody titers do not have a beneficial effect or even associate with an increased risk of recurrent wheezing (7–11). It is important to note that the antibody titers in these studies are determined by *in vitro* binding or neutralization assays, while additional antibody effector functions are not taken into account.

For nearly all licensed vaccines, antibodies are the presumed correlate of protection, but the underlying mechanisms of protection often remain unknown (12). Recent research suggests that, in addition to binding and neutralization, antibody effector functions are important contributors to protective immunity against several viruses, including influenza virus (13–15), HIV (16, 17), and Ebola virus (18, 19).

In contrast to their beneficial role in providing protection against infection and disease, antibodies have also been implicated in disease enhancement. For example, non-neutralizing dengue-specific antibodies have been shown to mediate antibody-dependent enhancement (ADE) of disease (20, 21). Interestingly, the 1960's formalin-inactivated (FI) RSV vaccine induced poorly-neutralizing antibodies which have been suggested to be involved in vaccine-enhanced disease upon natural infection (22–24). These examples illustrate the possibility that virus-specific antibodies contribute to pathogenesis when failing to protect.

Currently, the RSV field lacks a comprehensive overview of antibody effector functions in the context of RSV infection and disease. Here, we review what is known about various antibody effector functions during RSV infection, discuss their potential role in establishing the balance between protection and pathogenesis, and point out important gaps in our understanding of these processes. Moreover, we elaborate on the regulation of these effector functions on both the cellular and humoral side. Finally, we discuss the implications of antibody-mediated effector functions for the rational design of safe and effective vaccines and monoclonal antibody therapies against RSV. A thorough understanding of the role of antibodies in protection or disease during RSV infection is crucial for the development of new and improved vaccination strategies and may provide much-needed new insights into the precise mechanisms of antibody-mediated protective immunity.

## Fc-Mediated Antibody Effector Functions

**Figure 1:**
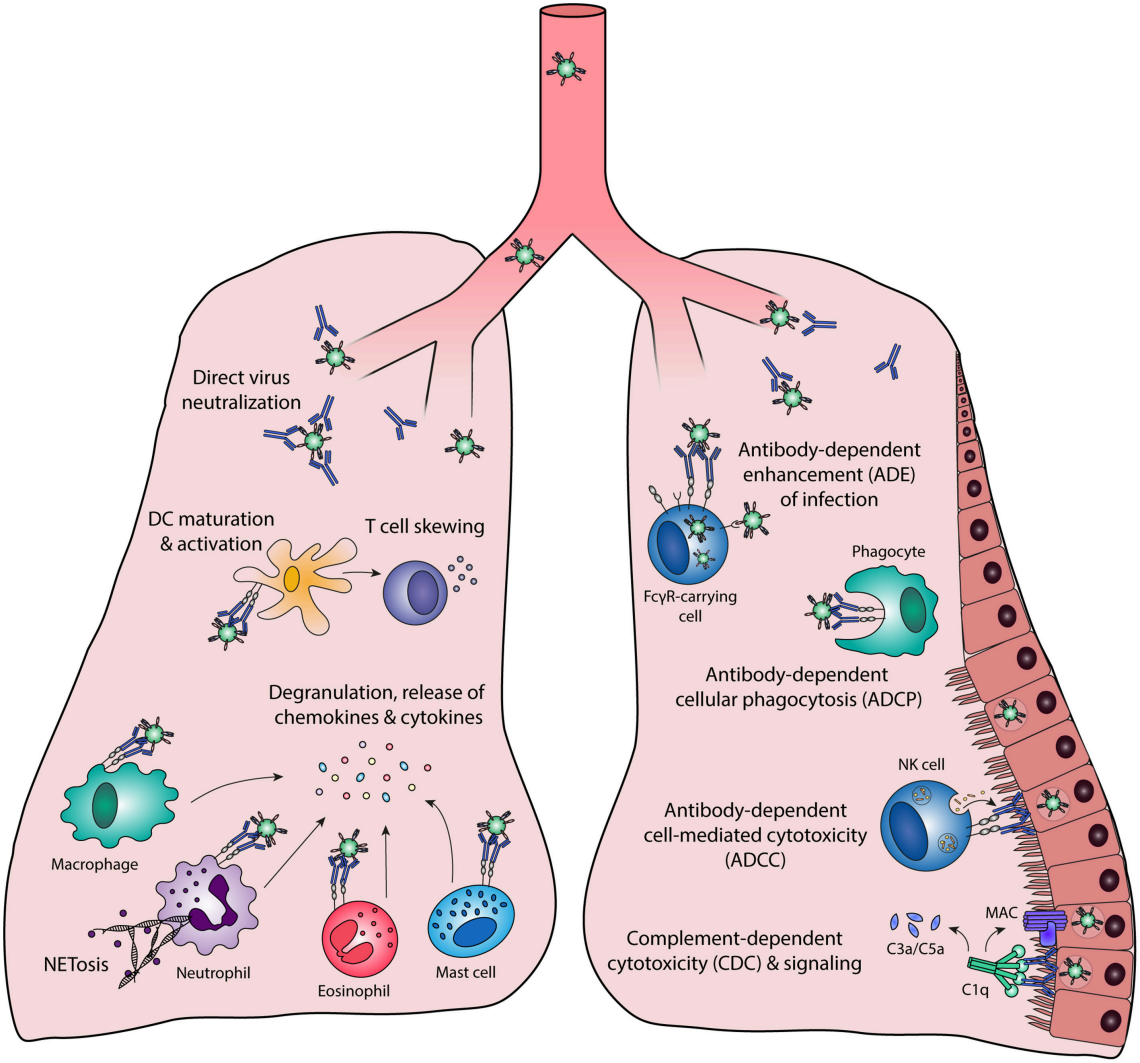
Fc-mediated antibody effector functions. Antibodies elicit a wide range of effector functions during viral infections. These include but are not necessarily limited to the functions depicted in this figure. DC, dendritic cell; FcγR, Fc gamma receptor; MAC, membrane attack complex; NK cell, natural killer cell.

Antibody effector functions are an important part of the humoral immune response and form an essential link between innate and adaptive immunity. Most of these effector functions are induced via the constant (Fc) region of the antibody, which can interact with complement proteins and specialized Fc-receptors. The latter can induce activating or inhibitory pathways, depending on the type of receptor, and are found on B cells and most innate immune cells in various combinations. The most well-known Fc-mediated antibody effector functions are antibody-dependent cell-mediated cytotoxicity (ADCC), antibody-dependent cellular phagocytosis (ADCP), and complement-dependent cytotoxicity (CDC). In addition, antibodies have been found to mediate inflammation and immunomodulation through the induction of cellular differentiation and activation. Each of these functions is described in detail below and a schematic overview is depicted in Figure 1.

## Antibody-Dependent Cell-Mediated Cytotoxicity (ADCC)

**Figure 2:**
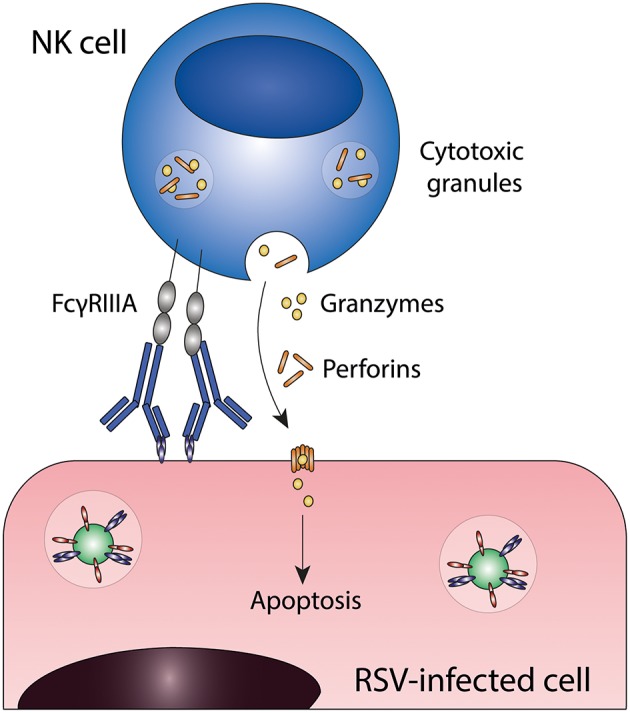
Antibody-dependent cell-mediated cytotoxicity (ADCC). Fc gamma receptors present on for example natural killer (NK) cells engage antibody-bound infected cells and induce target cell death through the release of cytotoxic granules. FcγRIIIA, Fc gamma receptor IIIA; NK cell, natural killer cell; RSV, respiratory syncytial virus.

ADCC is induced when Fc gamma receptors (FcγRs) on innate effector cells are engaged by the Fc domain of antibodies that are bound to viral proteins on the surface of virus-infected cells. This interaction induces the release of cytotoxic granules (containing perforins and granzymes) resulting in killing of infected cells (25). Multiple innate effector cells, including natural killer (NK) cells, neutrophils, monocytes, and macrophages, are capable of ADCC *in vitro*. However, the most important contributors to ADCC *in vivo* are thought to be NK cells, which express only FcγRIIIA. Figure 2 shows a schematic representation of ADCC.

In the field of tumor immunology, ADCC has been recognized as an important mechanism of action for therapeutic monoclonal antibodies (mAbs) that target tumor cells [as reviewed by (26)]. For infectious diseases, ADCC only recently started to gain attention. ADCC has been shown to form a critical component of effective immunity against HIV and influenza virus. ADCC-inducing HIV-specific antibodies were identified as a key correlate of protection in the RV144 HIV vaccine trial (27–29). Moreover, HIV-infected individuals who control the virus without antiretroviral therapy demonstrated a broader polyfunctional humoral immune response including ADCC activity compared to viremic individuals (30–33). There has been much debate about the role of ADCC during influenza-induced disease. Some studies point to the protective capacity of ADCC-inducing antibodies (34, 35), whereas others do not show any role for NK cells in antibody-mediated protection (36), or even suggest involvement of ADCC in exaggeration of the immune response (37–39). For multiple other clinically important viral infections, including dengue virus and Ebola virus, research into the effect of ADCC is ongoing (40–42). Taken together, ADCC seems to be involved in the immune response against multiple viruses and is therefore potentially of interest in the context of RSV infection.

### ADCC in RSV Infection

NK cells are the most important contributors to ADCC *in vivo* and important effector cells during RSV infection. In mice, increased numbers of NK cells are present in the lungs early after RSV infection (43–45). In RSV-infected infants, the proportion of NK cells has been reported both to be decreased (46–49) or increased (50, 51) in comparison with healthy controls or infants with mild symptoms. Since maternally-derived antibodies are virtually always present during primary RSV infection and antibody-coated virus-infected cells are a trigger for ADCC, it can be assumed that ADCC occurs during primary RSV infection.

Although NK cells are thought to be the most important mediators of ADCC against virus-infected cells, this has never been shown for RSV. All studies mentioned below are performed with peripheral blood mononuclear cells (PBMCs), without distinction between different cell types. RSV-specific immunoglobulin G (IgG) has been shown to induce ADCC toward RSV-infected epithelial cells *in vitro* (52, 53). The major surface antigens of RSV are the fusion (F) and the attachment (G) protein which are both required for infectivity *in vivo*. The RSV F protein has two conformational states: post-fusion (post-F) and pre-fusion (pre-F), of which the latter is a potent target for neutralization (54). Multiple studies show that anti-RSV G antibodies are efficient inducers of ADCC *in vitro* (55, 56), and the involvement of this process in virus clearance *in vivo* has been proposed (57, 58). In contrast, anti-RSV F antibodies do not efficiently induce ADCC *in vitro* (55), although it must be noted that no distinction between pre- and post-F antibodies was made and the ADCC potential could differ between the two functional states of the F protein.

Antibodies from breast milk, cord blood, and nasopharyngeal secretions and serum from RSV-infected infants show ADCC activity *in vitro* (52, 53, 59). This shows that the antibodies that are present *in vivo* are capable of eliciting ADCC activity *in vitro*. Two studies showed that the level of ADCC activity measured *in vitro* was independent of clinical symptoms and age, suggesting that ADCC is not a determining factor in the varying clinical manifestations of primary RSV infection (53, 59). Interestingly, the ADCC capacity of serum antibodies from RSV-infected infants rapidly declines over time, whereas the neutralization capacity remains more stable. If ADCC is important in protection against infection, this decline could partly explain the susceptibility to repeated infections throughout life.

Limited evidence is present on the occurrence of ADCC during RSV infection *in vivo*. The most convincing data is provided by mouse studies performed with anti-RSV G protein-specific Fab- or F(ab′)_2_ fragments lacking the complete Fc domain, or aglycosylated antibodies lacking the glycosylation site that is required for efficient FcγR and complement interactions (58, 60, 61). It was shown that Fab fragments of the 1812A2B anti-RSV G antibody and F(ab')_2_ fragments of the 131-2G anti-RSV G antibody do not reduce viral load, whereas the corresponding intact antibodies do confer protection (58, 60). The authors propose that virus clearance by the 131-2G antibody is mediated through ADCC, however, the involvement of other Fc-mediated effector functions in this study cannot be ruled out. In an attempt to ascertain the role of ADCC by NK cells in the protective mechanisms of the anti-RSV G antibody 18A2B2, SCID beige mice (which are deficient in NK cell activity) were passively immunized with the full antibody (60). In this study, the absence of NK cells had no effect on the protective capacity of 18A2B2, pointing to the involvement of other Fc effector functions. Further research is needed to study the exact role of ADCC for other mAbs and RSV-immune serum. Passive immunization with aglycosylated 1C2 anti-RSV G antibodies reduced virus titers significantly but were not as effective as wildtype antibodies, indicating that protection by the 1C2 antibody is mediated by both Fc-dependent and Fc-independent mechanisms (61). Although these studies highlight the importance of Fc-mediated antibody effector functions in protection against RSV infection in the case of these specific anti-RSV G mAbs, the role of ADCC in protection or pathogenesis during natural RSV infection remains to be determined.

## Antibody-Dependent Cellular Phagocytosis (ADCP)

**Figure 3:**
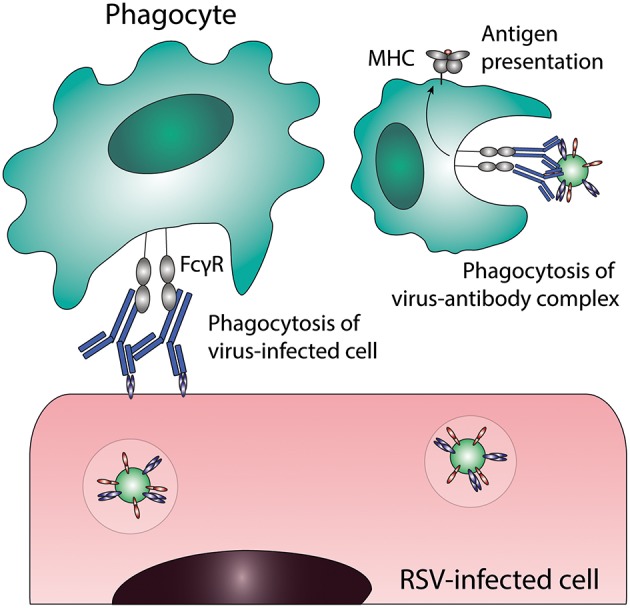
Antibody-dependent cellular phagocytosis (ADCP). Phagocytes can clear virus-infected cells and immune complexes that are engaged by Fc gamma receptors through phagocytosis. Uptake of viral particles or proteins leads to antigen presentation, which induces the adaptive immune system. FcγR, Fc gamma receptor; MHC, major histocompatibility complex; RSV, respiratory syncytial virus.

ADCP or opsonophagocytosis is the uptake of virus-antibody complexes or antibody-coated virus-infected cells by phagocytic cells (for a schematic representation of this process see Figure 3). Phagocytic cells, including monocytes, macrophages, neutrophils, eosinophils and dendritic cells (DCs), express FcγRI, FcγRII, and FcαRI, which can all mediate immune complex uptake. The exact phagocytic capacity of effector leukocytes is dependent upon the cell type, differentiation stage, and level of FcγR expression. ADCP results in the clearance of immune complexes from the infected host, by trafficking of the complexes to lysosomes for degradation and antigen processing for presentation on Major Histocompatibility Complex (MHC)-molecules on the cell surface. Interestingly, some viruses have exploited this mechanism to infect phagocytes by escaping from lysosomal degradation (described below in “Antibody-dependent enhancement of infection”).

ADCP has been extensively described for its role in protection against bacteria, but its importance during viral infections is unclear. Some studies have been performed for influenza virus, showing that phagocytosis by (alveolar) macrophages may contribute to protection from infection in mice (36, 62) and potentially plays a role in the recovery from severe infections in humans (15, 63). Also for cytomegalovirus (CMV), it was shown that vaccine-induced antibodies play an important role in vaccine efficacy, independent of neutralization or ADCC capacity (64). In accordance with these results, a study by Nelson et al. showed no role for neutralization or ADCC, while robust ADCP induction was observed (65). Antibody-mediated clearance by phagocytes *in vivo* has also been suggested for HIV (66, 67), adenovirus (68), West Nile Virus (WNV) (69), and foot-and-mouth disease virus (70, 71).

### ADCP in RSV Infection

Phagocytosis of RSV-antibody complexes or RSV-infected cells has to our knowledge never been directly explored as a protective immune mechanism for RSV. *In vitro* studies show phagocytosis of RSV immune complexes by neutrophils (56, 72, 73) and eosinophils (74). Varying levels of phagocytic activity have been observed for different RSV-specific monoclonal antibodies, suggesting that ADCP activity depends on epitope and/or affinity (56, 73). An *in vivo* mouse study has shown that macrophages are essential in conferring antibody-mediated restriction of RSV replication, whereas neutrophil depletion did not significantly affect pulmonary viral replication (75). This suggests that Fc-mediated effector functions executed by macrophages rather than neutrophils are important in protection against RSV infection in a mouse model.

Besides the uptake of viral particles, phagocytosis initiates the activation of cells. This can result in the release of a broad range of effector molecules (72–74), which will be described in detail in “Antibody-dependent immunomodulation during RSV infection.” Although there is limited evidence on the role of ADCP during RSV infection, the importance of macrophages in antibody-mediated protection in mice provides a basis for further investigation.

## Antibody-Mediated Complement Activation

**Figure 4:**
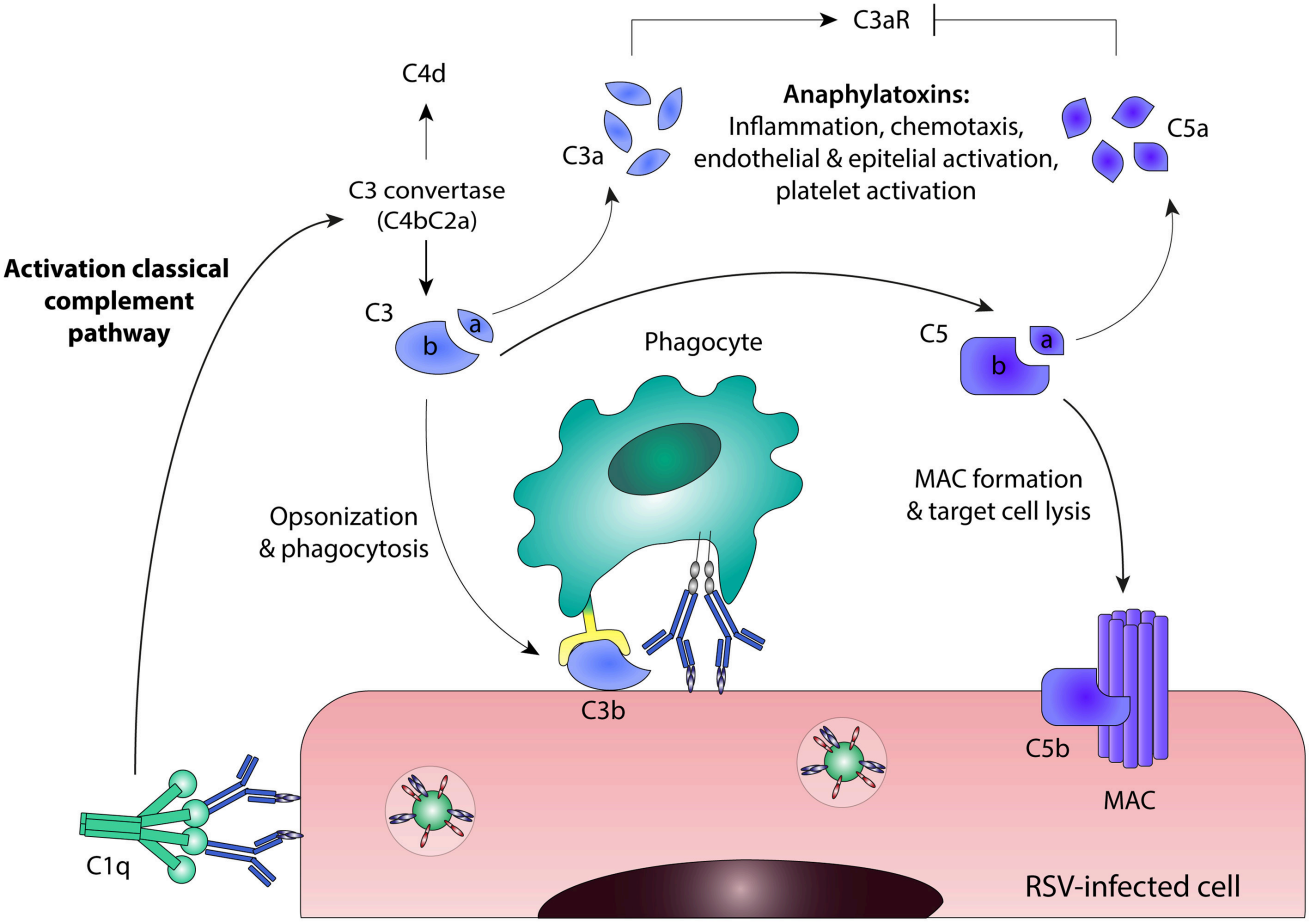
Antibody-mediated complement activation. Binding of C1q to antibody-bound virus-infected cells leads to activation of the classical complement pathway. C3 convertase (C4bC2a) is formed and cleaves C3 into C3a and C3b. Active C4b can be cleaved into the enzymatically inactive form C4d, which serves as a marker for complement activation. Further downstream in the classical complement pathway, C5 is cleaved into C5a and C5b. C3a and C5a are anaphylatoxins that stimulate a pro-inflammatory environment, although they act in different ways: C3a induces C3aR signaling, whereas C5a inhibits C3aR expression. C3b binds to pathogens and infected cells, leading to phagocytosis through complement receptors found on immune cells. The terminal complement components will assemble into the membrane attack complex (MAC), resulting in direct lysis of the infected cell. C3aR, C3a Receptor; MAC, membrane attack complex; RSV, respiratory syncytial virus.

Besides ADCC and ADCP, antibodies can also induce complement activation. The complement cascade contributes to pathogen elimination either directly, by means of complement-dependent cytotoxicity (CDC), or indirectly, through phagocytic clearance of complement-coated targets and the induction of an inflammatory response. Activation of the classical complement pathway results from binding of the recognition molecule C1q to the Fc domain of antibodies bound to virus-infected cells (76, 77), as depicted in Figure 4. Upon binding of C1q, the proteases of the classical pathway are activated, leading to cleavage of C2 and C4. Together, the resulting cleavage products form the C3 convertase (C4bC2a) that cleaves C3 into C3a and C3b. One of the mechanisms by which the complement cascade is regulated, is cleavage of active C4b, which serves as a marker for complement activation. The release of anaphylatoxins C3a and C5a stimulates a pro-inflammatory environment by inducing the recruitment of immune effector cells and the activation of leukocytes, endothelial cells, epithelial cells, and platelets (78, 79). The highly reactive C3b binds to pathogens and infected cells, leading to immune complex clearance and phagocytosis through complement receptors found on immune cells. The terminal complement components will assemble into the membrane attack complex (MAC), resulting in lysis of the infected cell. Besides direct antiviral activity, the complement system can also regulate B cell responses. The binding of complement-coated immune complexes to complement receptor 2 on B cells is reported to lower the B cell activation threshold, thereby promoting long-lived adaptive immunity and higher antibody levels (80, 81).

Complement can have both a protective and pathogenic role during viral infections. The protective capacity of poorly neutralizing antibodies during WNV infection is mediated by the complement system, as was shown using knockout mice (69). The presence of complement even enhances the neutralization capacity of WNV-specific antibodies (82). In addition, an important role for complement has been shown in the protective capacity of (monoclonal) antibodies against influenza virus (38, 83), vaccinia virus (84), CMV (85), and HIV (66, 67). In contrast, complement activation has also been suggested to contribute to disease severity in dengue virus (86, 87) and HIV infection (88, 89).

### Antibody-Mediated Complement Activation in RSV Infection

The complement system consists of multiple components and elicits its effector functions through different pathways. Early studies have shown antibody and complement deposition on nasopharyngeal cells of RSV-infected infants (90). Whether this contributed to viral clearance or disease was not determined. Studies in complement-deficient mice have shown that complement is important in antibody-mediated protection against RSV infection (60, 75). A number of different mechanisms have been suggested for this complement-enhanced protection. Firstly, direct enhancement of the neutralization capacity of antibodies by fixation of complement components to virus-antibody complexes may increase the steric hindrance of bound antibodies (91). Another mechanism that could be at play is complement-dependent opsonization of virus-infected cells, which leads to subsequent uptake by phagocytes. Finally, complement has also been shown to increase the CD4(+) T cell response in the presence of RSV immune serum in an *in vivo* mouse model (92).

Besides its potential role in the clearance and/or pathogenesis of natural RSV infection, complement activation has been suggested to contribute to disease enhancement induced by natural infection following FI-RSV vaccination. C3a receptor (C3aR)-deficient mice had decreased airway hyperresponsiveness (AHR) and less mucus production in an FI-RSV vaccination-challenge model (93). In this study, C3aR expression was enhanced in C5-knockout mice, showing that the balance in activation of different complement factors (C3a vs. C5a) is important in determining disease outcome. Moreover, Polack et al. demonstrated the co-localization of IgG and C3 in the lungs of mice with enhanced RSV disease, but not in control mice (22). In addition, both C3- and B cell-knockout mice showed a decrease in bronchoconstriction compared to WT mice vaccinated with FI-RSV. Therefore, in a mouse model of vaccine-enhanced disease, the presence of C5 seems protective, whereas C3a promotes enhanced disease. This is also supported by the limited data available on complement activation during vaccine-enhanced disease in infants. Lung sections of the two children who died of vaccine-enhanced disease had extensive deposition of complement cleavage product C4d, which serves as a stable marker for complement activation (22). The presence of C4d provides evidence for complement activation during vaccine-enhanced disease in infants, but it remains to be determined whether there is a causal relation between complement activation and vaccine-enhanced disease. Finally, mouse studies point to the involvement of complement components in the development of AHR and asthma upon RSV infection (94, 95). Taken together, the complement system seems to be important in antibody-dependent protection *in vivo*, but it also potentially contributes to (vaccine-enhanced) disease and asthma, suggesting a dual role in RSV infection that requires further investigation.

## Antibody-Mediated Immunomodulation

**Figure 5:**
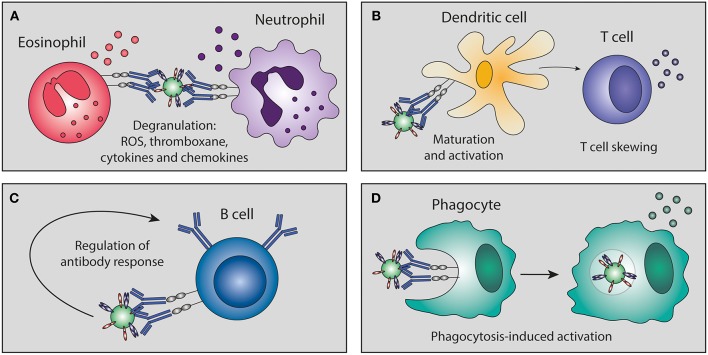
Antibody-mediated immunomodulation. Immune complexes can skew immune cell maturation and activation of granulocytes, dendritic cells, T cells, B cells, and phagocytes. This immunomodulation can lead to **(A)** degranulation, **(B)** skewing of T cell responses, **(C)** regulation of B cell antibody responses, and **(D)** phagocytosis-induced secretion of immunomodulatory mediators. ROS, reactive oxygen species.

Besides the well-defined classical Fc-mediated effector functions (ADCC, ADCP, CDC), immune complexes can also promote immune cell maturation and activation, leading to a wide range of effector activities and production of pro-inflammatory and immunomodulatory mediators (a limited overview is depicted in Figure 5). Some of these pro-inflammatory cytokine responses correlate with protection as has been shown for influenza (62) and HIV (96). The importance of FcγRs in this process has been shown by the use of FcγR-deficient mice [as extensively reviewed in (97)]. In contrast to the pro-inflammatory responses caused by immune complexes, injection with intravenous immunoglobulin (IVIg) can induce an anti-inflammatory state. It is proposed that this anti-inflammatory effect is partly due to the presence of sialylated antibodies in IVIg, which induce expression of FcγRIIB (the only inhibitory FcγR) and thereby dampen the inflammatory response (98).

Immune complexes can also regulate cellular maturation and activation. The balance between inhibitory and activating FcγR interactions is crucial in regulating B cell IgG responses (99–101), and skewing APC maturation and antigen presentation (102–105), which can modulate T cell activation. Immune complexes have also been shown to bias the macrophage immune response toward a Th2-like phenotype (106).

### Antibody-Mediated Immunomodulation in RSV Infection

RSV-antibody complexes can lead to activation of phagocytes either directly or after phagocytosis, resulting in the production of reactive oxygen species (ROS), thromboxane, (pro-inflammatory) cytokines, and chemokines (72, 73, 107), which may contribute to viral clearance. However, these mediators can also have immunopathological effects, including tissue damage, platelet aggregation, and bronchoconstriction. Given that neutrophils are the predominant airway leukocytes present in RSV-infected infants, their activation is suggested to be involved in the induction of severe RSV disease (108). Interestingly, in contrast to RSV immune complexes, it has been reported that RSV alone does not lead to ROS production by granulocytes (107) and can even inhibit this process (73, 109). It has been suggested that anti-RSV G mAbs are less potent inducers of ROS and cytokine production than anti-RSV F mAbs (73), but this was based on experiments with only two RSV-specific antibodies. Notably, differences in the capacity to induce a response may not be due to antigen-specificity *per se* but rather due to epitope localization, as described in the paragraph “Important epitopes in RSV infection.”

Excessive eosinophilic activation has been suggested to play a role in the immunopathology of FI-RSV-induced disease in mice (22). Whether the non-neutralizing antibodies induced by the FI-RSV vaccine play a role in this activation remains unknown. *In vitro* studies have shown that eosinophils can phagocytose RSV-antibody complexes, leading to degranulation (74). The use of heat-inactivated serum abolished this effect, indicating complement involvement.

Besides an immunomodulatory effect on granulocytes, RSV-antibody complexes can also affect T cell responses. Kruijsen et al. show in an *in vivo* mouse model that IFN-γ secretion by CD4(+) T cells is increased in the presence of RSV immune serum (92). This increase is dependent on both FcγRs and the complement system. Additional *in vitro* experiments indicate that both anti-RSV G, as well as anti-RSV F antibodies can induce this enhanced CD4(+) T cell response, whereas CD8(+) T cells are only activated by the presence of anti-RSV G antibodies. Another *in vitro* study found that DCs primed with complexes composed of RSV and F-specific antibodies displayed an impaired capacity to activate CD8(+) and CD4(+) T cells (110).

RSV-antibody complexes also contribute to antibody-mediated immunomodulation through the induction or inhibition of cytokine and chemokine production in PBMCs. In an *in vitro* study, RSV-antibody complexes inhibited IFN-α production in PBMCs, whereas these complexes enhanced IFN-α production of PBMCs in the absence of CD14(+) cells (111). Another *in vitro* study showed that, compared to RSV alone, RSV immune complexes induce increased IFN-α, IFN-γ, CXCL10, and CXCL11 production in monocytes (112). In infant PBMCs, only CXCL10 production was significantly enhanced. CXCL10 can mediate a neutrophil-dependent excessive pulmonary inflammation (113), which could contribute to RSV pathogenesis. This indicates that immune complexes can potentially also activate neutrophils indirectly, through the induction of chemokines and cytokines in PBMCs. Altogether, these studies show that immune complexes are able to skew the RSV-specific immune response in multiple ways, but more research is needed to clarify the exact contribution of antibody-mediated immunomodulation to protection and disease during RSV infection.

## Antibody-Dependent Enhancement (ADE) of Infection

**Figure 6:**
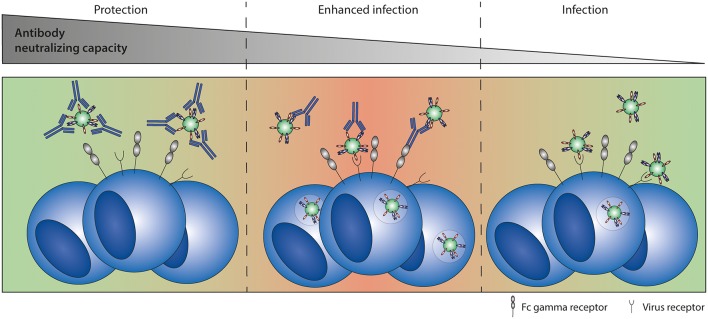
Antibody-dependent enhancement (ADE) of infection. ADE of infection has been shown *in vitro* for multiple viruses, including RSV. High antibody titers neutralize the virus completely. Sub-neutralizing antibody titers form immune complexes that can interact with both the virus receptor and Fc gamma receptors, leading to enhanced infection levels compared to infection in the absence of antibodies.

ADE refers to a phenomenon in which virus-specific antibodies promote, rather than inhibit, infection and/or disease. In ADE of infection, also known as extrinsic ADE (114), the number of virus-infected cells is increased in the presence of (natural or monoclonal) antibodies that are non-neutralizing or present in sub-neutralizing concentrations. ADE of infection requires the presence of FcγRs on target cells and is an efficient *in vitro* tool to assess Fc-FcγR interactions. However, while ADE of infection has been observed for many viruses *in vitro* [as extensively reviewed in (115)], its significance *in vivo* remains uncertain. A schematic representation of ADE of infection is depicted in Figure 6.

### ADE of RSV Infection

ADE of RSV infection has been demonstrated *in vitro* for both mAbs and RSV-immune serum in monocytic cell lines, PBMCs, neonatal, and adult NK cells, and primary mouse and cotton rat immune cells (110, 116–120). However, whether the ADE of infection observed *in vitro* is related to *in vivo* disease outcome is doubtful. No correlation has been found between disease severity in infants and the capacity of serum antibodies to induce ADE of RSV infection *in vitro* (119). Furthermore, ADE of infection has never been demonstrated *in vivo*. However, it must be noted that this has never been assessed during FI-RSV vaccine-enhanced disease.

## Antibody-Dependent Enhancement (ADE) of Disease

**Figure 7:**
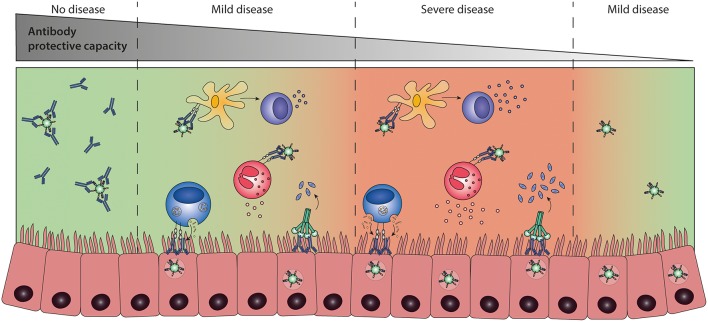
Antibody-dependent enhancement (ADE) of disease. ADE of disease refers to a process in which the presence of pathogen-specific antibodies contributes to disease severity. Highly neutralizing antibodies result in sterile immunity, preventing infection and disease (left panel). The presence of low levels of protective antibodies allows for viral replication and leads to an Fc-mediated immune response that can either contribute to protection (second panel) or potentially lead to more severe disease (third panel) compared to infection in the absence of antibodies (right panel). It is currently unknown whether Fc-mediated effector functions can lead to severe disease and which immunological mechanisms determine the difference between protective or enhancing Fc-mediated responses.

ADE of disease, or instrinsic ADE (114), refers to a process in which the presence of pathogen-specific antibodies contributes to disease severity. For example, immune complexes might bind to FcγR-expressing immune cells, modulating the immune response, and subsequently leading to enhanced inflammation. ADE of disease has been a presumed cause of severe disease following various viral infections and vaccinations (37, 114, 121–123). However, the underlying mechanisms are largely unknown and *in vivo* data supporting these claims are often lacking. However, for dengue virus infection some first clues to unravel the mechanism underlying ADE of disease have recently been published. Wang et al. have been able to show a correlation between FcγRIIIA binding capacity of dengue virus antibodies and disease severity *in vivo* (21). The dengue-specific antibodies are thought to cross-react with platelet antigens and induce thrombocytopenia. Suggested underlying mechanisms are FcγR-mediated platelet activation, phagocytosis, or ADCC, but further investigation is needed to confirm these hypotheses. In addition, Katzelnick et al. have shown that high dengue-specific antibody titers correlate with protection, whereas intermediate antibody titers correlate with severe dengue disease (124). Although low or no antibody titers are not protective, they do not enhance disease. It is possible that RSV-specific antibodies show a similar pattern, as illustrated schematically in Figure 7.

### ADE of RSV Disease

Although *in vitro* ADE of infection does not seem to be a determinant for severe RSV disease (119), other antibody-mediated mechanisms could be involved, as has recently been shown for dengue virus infection (21). Many animal studies on RSV infection highlight the role of an excessive immune response in FI-RSV vaccine-enhanced disease. It is likely that poorly-neutralizing vaccine-induced antibodies play a role in the development of FI-RSV vaccine-enhanced disease (22–24), although it remains uncertain which Fc-mediated effector functions are involved.

Little is known on the involvement of (maternal) antibodies in the development of severe disease after natural RSV infection. Severe RSV infections are most frequently seen in the first 6 months of life when infants have circulating maternal RSV-specific antibodies (2). This suggests that RSV-specific antibodies may contribute to the induction of severe RSV disease. Results from animal studies with Fab fragments and FcγR-knockout mice indeed show the involvement of antibody-mediated effector functions both in protection against viral replication (58, 60, 61) and in promoting inflammation (92).

Some studies have reported enhanced RSV disease to occur in the presence of waning immunity. Murphy et al. reported enhanced pulmonary pathology 3 months after immunization with a RSV F glycoprotein vaccine (125), which was not seen 1 week after immunization (126). In a follow-up study, enhanced lung pathology was observed upon immunization with low doses of recombinant F protein, mimicking waning immunity (127). Interestingly, the enhanced disease was independent of the presence of a Th1- or Th2-biasing adjuvant.

Taken together, there are clear indications suggesting that Fc-mediated antibody effector functions may contribute to severe RSV disease. Complement activation has been linked to vaccine-enhanced disease and asthma, and may therefore also be involved in severe RSV disease upon natural infection. In addition, the immunomodulatory effects of immune complexes can lead to a pro-inflammatory environment, which is thought to be the underlying cause of RSV-mediated pathology. However, more research on the involvement of individual Fc-mediated effector functions in disease outcome following RSV infection is needed.

## Regulation of Fc-Mediated Effector Functions

Fc-mediated antibody effector functions play an important role in shaping the immune response and their active regulation is crucial to prevent excessive immune activation. A number of determinants have been found to influence Fc-mediated effector functions on both the cellular and antibody side of the Fc-Fc receptor (FcR) interaction. Important antibody characteristics are the isotype, subclass, glycosylation pattern, and antigen specificity, while important cellular determinants are the epitope position relative to the target cell membrane and FcR expression and polymorphisms on the effector cell, which together determine the capacity of the antibody to interact with specific FcRs. Most antibodies are not specifically eliciting a single effector function, and therefore the combination of all these characteristics determines the outcome of the various Fc-FcR interactions and the interaction with the complement system.

## Antibody Isotype and Subclass

Antibodies consist of two functional domains: the variable antigen-binding fragment (Fab) and the constant fragment (Fc) that interacts with FcRs and C1q. The isotype of the Fc domain (IgA, IgD, IgE, IgG, and IgM) represents the major determinant of Fc-mediated effector functions. Of these isotypes, IgG is the most important when it comes to Fc-mediated effector functions, as this is the only isotype known to interact with the widely expressed FcγRs. Whereas, the majority of antibodies in serum are of the IgG subtype, IgA is the major isotype present in mucosal secretions. This isotype interacts with its specific receptor FcαRI, which is present on neutrophils, eosinophils, monocytes, and macrophages [extensively reviewed in (128)]. Activation of FcαRI by IgA-opsonized pathogens can induce ADCC, phagocytosis, degranulation, and cytokine release. Other important isotypes to briefly mention are IgM, which is a potent complement activator (76), and IgE, which has been linked to various allergic diseases.

In humans, four different IgG subclasses (IgG1-IgG4) are known. These subclasses differ in amino acid sequence, which influences their capacity to interact with certain classes of FcγRs and complement components as depicted in Table 1. Production of different isotypes and subclasses is tightly regulated and dependent on differentiation of the B cell, which can be influenced by cytokines and interactions with pattern-recognition receptors. The response to protein antigens usually involves T cell help and induces class switching to IgG1 or IgG3, whereas polysaccharide antigens induce class switching to IgG2 in the absence of T cell help (132). Viral infections, including RSV infections, mostly induce IgG1 and IgG3 antibody responses (133–135).

**Table 1:** Binding capacity and functionality of IgG subclasses.

**Subclass**	**Serum abundance (%)**	**FcγRI**	**FcγRIIa**	**FcγRIIb**	**FcγRIIIa**	**FcγRIIIb**	**C1q**	**Effector functions**
IgG1	60	+++	+++	+	++	+++	++	ADCC, ADCP, CDC
IgG2	32	–	++	–	–	–	+	
IgG3	4	++++	++++	++	++++	++++	+++	ADCC, ADCP, CDC
IgG4	4	++	++	+	–	–	–	

*(129–131). ADCC, antibody-dependent cell-mediated cytotoxicity; ADCP, antibody-dependent cellular phagocytosis; CDC, complement-dependent cytotoxicity; FcγR, Fc gamma receptor; IgG, immunoglobulin G*.

IgG1 and IgG3 have the highest affinity for FcγRs and are potent activators of complement, ADCC and phagocytosis (129, 136, 137). IgG3 is the subclass with the highest potential to activate both FcγRs and complement, but due to its short half-life the preferred subclass for therapeutic cytotoxic activity is IgG1 (138). In contrast, receptor-blocking antibodies are often of the IgG2 or IgG4 subclass to avoid Fc-mediated cytotoxic side effects (139). Induction of specific subclasses can have major effects on the outcome of vaccine trials as has been shown for the HIV RV144 and VAX003 vaccines. RV144 recipients produced highly functional IgG3 antibodies that provided 31.2% efficacy, whereas VAX003 recipients elicited a monofunctional IgG4 antibody response that was not protective at all (140).

### Antibody Isotype and Subclass in RSV Infection

Severe RSV-mediated disease is most prevalent in infants below 6 months of age. These children mainly rely on maternally-derived IgG for protection against infectious diseases, but the correlation between serum IgG levels and protection against RSV disease is poor (7, 9–11). A recent study by Habibi et al. found that mucosal IgA titers are a better predictor of susceptibility to RSV infection than serum IgG levels in an adult challenge model (141). In addition, they showed a hampered IgA memory B cell response to RSV, which may explain the life-long susceptibility to repeated RSV infections. In accordance with these results, lower levels of nasal IgA were found in naturally RSV-infected adults compared to healthy controls (142). These findings highlight the importance of mucosal IgA in protection against RSV infection. However, it is questionable whether IgA plays a role in protection or disease during primary infection. IgA antibodies to RSV are only found in secretions after 4 months of age, confirming they are synthesized as a result of (primary) infection (143). RSV-specific IgA has been shown to induce antibody-mediated effector functions. Although palivizumab-IgA demonstrated slightly higher lysis of RSV-infected HEp2 cells by neutrophils (but not monocytes) *in vitro*, there was a somewhat decreased efficacy *in vivo* compared to palivizumab-IgG (144). Additional experiments with FcαRI transgenic mice suggest that IgA-mediated protection is Fc receptor-independent. No further research with RSV-IgA immune complexes has been published to date and therefore their role in protection or disease remains to be investigated.

Another interesting isotype is IgE, as the results from multiple studies suggest the involvement of this isotype in the development of RSV-mediated bronchiolitis and wheezing (145–148). In a mouse model, RSV-specific IgE has been shown to enhance airway hyperresponsiveness (149). Since all infants produce IgE in response to RSV infection (150), it is thought that the height and duration of the IgE response are important for the induction of subsequent immunopathology (148, 151, 152). Mast cells abundantly express the IgE-specific Fc receptor (FcεRI) and were shown to play an important role in IgE-induced airway hyperresponsiveness in an RSV reinfection mouse model (149).

In addition to studies on isotypes, extensive studies have been performed on the presence of IgG subclasses during RSV infection. Wagner et al. have performed some early studies into the antibody subclass response to the RSV F and G glycoproteins in both infants and adults (133, 153, 154). Primary RSV infections predominantly gave rise to IgG1 and IgG3 antibodies, whereas subsequent infection only led to an increase in IgG1 and IgG2 titers (133). RSV infection led to a poor IgG4 antibody response in all subjects. RSV F protein was the most immunogenic, leading to higher antibody titers compared to the RSV G protein (154). The IgG1/IgG2 ratio of antibody titers to the RSV F protein was fourfold higher than to the RSV G protein after the first three RSV infections in infants. This difference was thought to be due to the extensive glycosylation of the G protein, resulting in IgG2 antibodies. IgG1 and IgG3 are the most potent FcγR-binding subclasses. This suggests that the majority of anti-RSV F antibodies are effective inducers of Fc-mediated effector functions, in contrast to the IgG2 subset of anti-RSV G antibodies. Experimental RSV infection in adults showed similar subclass responses to RSV F and G protein (153). A recent study confirms the findings of Wagner et al. showing a strong IgG3 response in infants younger than 4 months, despite the presence of high levels of maternal antibodies (155). A rise in RSV-specific IgG1 and IgG2 was only observed in infants older than 7 months.

Besides human studies, several mouse studies have been performed to investigate the subclass antibody response. Although some homology between mouse and human IgG subclasses has been found, it is unclear whether they induce the same downstream immune responses. In mice, neonatal IgG responses to RSV infection are significantly skewed toward mIgG1 (homologous to human IgG4), indicating a Th2 bias (156), whereas primary infection in adult mice leads to a balanced mIgG2a/mIgG1 response (homologous to human IgG1/IgG4) (157). Compared to wild-type RSV infection, immunization with inactivated or non-replicative RSV led to a low mIgG2a/mIgG1 ratio (24, 158). The largest proportion of antibodies directed at the RSV-F protein was mIgG2 (homologous to human IgG1), whereas the G protein response had a significantly lower proportion of mIgG2 (158). These results indicate that both the age of the host and the antigens determine the subclass response. However, it is remarkable that RSV infection leads to a poor IgG4 antibody response in humans, but to a high mIgG1 (homolog of human IgG4) response in (neonatal) mice. Thus, caution is warranted in the translation between human and mouse antibody studies.

Although extensive studies have been performed on the presence of specific subclasses, evidence on the role of these different subclasses during RSV infection is limited. One study describes a direct comparison between the functionality of palivizumab-IgG1 and -IgG2 (159). The neutralizing potential of both subclasses was comparable. However, the IgG2 antibody showed negligible binding to murine FcγRs and human C1q, resulting in less efficacy *in vivo* as measured by increased viral lung titers in challenged cotton rats (159). This finding underscores the protective potential of IgG1-mediated effector functions during RSV infection.

## Antibody Glycosylation

**Figure 8:**
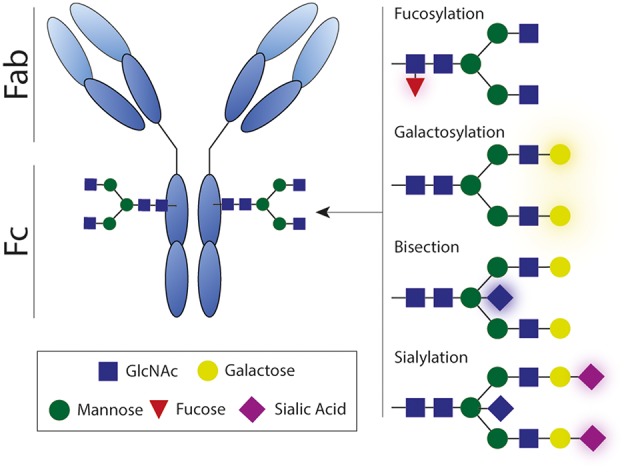
Antibody glycosylation. Each IgG molecule contains a glycosylation site that can harbor a variety of glycans, consisting of varying combinations of mannose, (bisecting) N-acetylglycosamine, fucose, galactose, and sialic acid. Antibody effector activity is substantially impaired in absence of this glycan. Fab, antigen-binding fragment; Fc, crystallizable fragment; GlcNAc, N-acetylglycosamine.

Glycosylation of the antibody Fc domain is another important regulator of Fc-mediated effector functions. Each IgG molecule contains a highly conserved asparagine at position 297 (N297) that functions as a glycosylation site that can harbor a variety of glycans, consisting of varying combinations of mannose, (bisecting) N-acetylglycosamine (GlcNAc), fucose, galactose, and sialic acid (Figure 8). The complete absence of this glycan leads to a conformational state that is non-permissive for FcγR or complement binding, thereby impairing Fc-mediated antibody effector functions.

Afucosylation has the most straightforward influence on antibody effector functions. The absence of the core fucose on the Fc-glycan directly boosts ADCC activity by enhancing the interaction with FcγRIIIA (Figure 2) (160–162). Interestingly, afucosylated mAbs have shown to be more protective against various infectious agents (163, 164) and more efficacious in cancer therapy (165, 166). However, increased levels of afucosylation are also associated with severe disease during secondary dengue infection (21).

Another biologically important modification to the Fc glycan is sialylation. The presence of sialic acid inhibits FcγR binding and is reported to be partly responsible for the anti-inflammatory activity of IVIg (98, 167). Besides having anti-inflammatory properties, sialylated Fc glycans have also been shown to induce the production of high-affinity broadly neutralizing antibodies against influenza virus (101).

Besides its effect on Fc receptor interactions, Fc glycosylation also affects complement C1q binding to immune complexes. A recent study shows that elevated galactosylation and sialylation increase C1q-binding, downstream complement deposition, and complement dependent cytotoxicity (168). In contrast, agalactosylated IgG has also been suggested to elicit enhanced complement activation, considering its role in several autoimmune diseases (169). These findings suggest that activation of complement potentially contributes to pathogen clearance, but can also contribute to inflammation in autoimmune disease, highlighting the dual role of complement.

Fc glycosylation is subject to active regulatory mechanisms that control the composition of the glycan structure. Major changes in glycosylation occur during pregnancy (170, 171), upon vaccination (101, 172), and during certain viral infections (101). Therefore, insight in the glycosylation pattern during RSV infection and disease may provide valuable clues on the cause of severe RSV disease.

### Antibody Glycosylation in RSV Infection

To our knowledge, only one group has investigated the effect of glycosylation in the response toward RSV infection. Hiatt et al. compared the original Palivizumab mAb with an afucosylated and agalactosylated plant-produced glycovariant (G0) in different *in vitro* and *in vivo* assays (159). The G0 glycovariant showed enhanced binding to murine FcγRs but less binding to human C1q compared to the parental Palivizumab, whereas neutralization capacity was comparable. The *in vivo* protective capacity of the G0 glycovariant was improved compared to the original, as evidenced by decreased pulmonary viral titers. In conclusion, this study suggests that the influence of Fc-glycosylation may be important in the protective capacity of RSV-specific antibodies but this needs to be studied in more detail for other mAbs and virus- and vaccine-induced antibodies.

## Epitope Position

Next to antibody structure and glycosylation, the location of the antibody-bound epitope with respect to the membrane of the infected cell has been shown to be pivotal in determining Fc-mediated effector functions. Since the use of mAbs, it has been noticed that different mAbs binding the same target protein can elicit different effector mechanisms (173). Antibodies binding to epitopes closer to the membrane (membrane proximal epitopes) mediate ADCC and CDC activity more efficiently, whereas antibodies that target membrane distal epitopes are often highly neutralizing and efficient ADCP-inducers (13, 174–176). More specifically, recent research suggests that ADCP is most efficiently triggered when antibodies bind within 10 nm from the cell surface (177), indicating that the optimal ADCP-inducing epitope is located neither too close, nor too far away from the cell membrane. These studies suggest that besides the common need for particular Fc-FcγR interactions, there are fundamental differences in the activation requirements of specific Fc-mediated effector functions. For CDC activity, stabilization of complement components on the cell surface is essential. This would require a short distance from epitope to cell membrane. During ADCC, the formation of an immune synapse is essential. This small synapse can only be formed when the NK cells engage antibodies bound in close proximity to the cell membrane, explaining the need for membrane proximal epitopes (175).

### Important Epitopes in RSV Infection

Neutralization of RSV is mainly established by antibodies against the RSV F and RSV G protein (178). Antibodies against the SH and N protein have also been described (179, 180) and although these antibodies are not involved in neutralization, they may have other important (Fc-mediated) functions (181). Capella et al. recently showed that antibodies against the pre-F protein were the most prevalent RSV-specific serum antibodies in infants below 2 years of age (182). Both serum IgG levels against anti-RSV pre-F and G correlated with disease severity in this study.

Various antigenic sites (named Ø and I-VIII) have been described for the two conformational states of the RSV F protein (183, 184). Pre-F-specific antibodies are better neutralizers than post-F-specific antibodies (185). However, not all pre-fusion F antibodies have similar neutralizing activity (183). The most potent neutralizing antibodies bind to distal epitopes, suggesting that the neutralizing potential of anti-RSV F antibodies not only relies on the conformation of F on which the epitope is present (e.g., pre- vs. post-F), but may also depend on the location of the epitope relative to the viral or cellular membrane. As described above, the proximity to the membrane determines the efficiency of Fc-mediated effector functions (13, 174, 175). This suggests that potently-neutralizing antibodies, binding to distal epitopes, may also be efficient inducers of ADCP. Antibodies binding to proximal epitopes are generally less neutralizing, but may be more potent in inducing ADCC and CDC.

The most important antigenic site for the RSV G protein is the central conserved domain (CCD). Despite the high variability of RSV G, antibodies against the CCD are broadly neutralizing against both RSV A and B strains (186). The G protein CCD binds to the CX3CR1 receptor, leading to attachment of RSV to its target cells (187). Antibodies against this receptor-binding domain efficiently neutralize RSV infection and decrease pathogenesis by binding soluble G protein, an immune evasion protein secreted by RSV-infected cells (56, 57, 188). Soluble G protein has been found to inhibit Fc-mediated antiviral effects of macrophages and complement (75), and to modulate trafficking of CX3CR1(+) cells (189). Next to the important roles mentioned above, antibodies against the CCD domain are also able to induce Fc-mediated effector functions like ADCP and ADCC (56).

Taken together, not only the antigen but also the epitope determines the efficacy of antibodies. Interestingly, evidence suggests that targeted epitopes may differ between infants and adults (190), but the effect of these changes on the efficacy of the antibody response is unknown. Further research may uncover the relation between antigenic site and effector functions against RSV infection, and thereby reveal preferred antibody-binding sites for protection against RSV disease.

## FcγR Expression and Polymorphisms

Another regulator of Fc-mediated effector functions is the expression pattern and polymorphisms of FcγRs. The majority of leukocytes express more than one FcγR type with varying downstream signaling activities. The level and variety of FcγR expression is tightly regulated during leukocyte development and can be modulated by certain mediators present during infection, inflammation, or even vaccination (103, 191). As stated before, the balance between inhibitory and activating FcγR interactions is crucial in regulating B cell IgG responses (99–101) and skewing APC maturation and antigen presentation (102–105). Additionally, co-engagement and signaling through other receptors such as TLRs may influence the activation threshold (192). Altogether, this points out the importance of receptor expression patterns on effector cells.

Besides variation in FcγR expression patterns, single nucleotide polymorphisms (SNPs) in FcγRs occur. Although many SNPs have been identified, only few have been shown to impact receptor function (193). One of the functional SNPs has been identified in FcγRIIa. Only the R131H allelic variant of this receptor is capable of interacting with IgG2, enabling efficient phagocytosis (194, 195). Another SNP affecting binding affinity has been characterized for FcγRIII, which has two co-dominantly expressed allotypes: V158 and F158. The presence of a valine residue at position 158 increases the affinity for IgG1 and IgG3, augmenting for example NK cell activity (196, 197).

### FcγR Expression and Polymorphisms in RSV Infection

Different FcγRs can have opposing effects on the immune response, as has also been shown for RSV. In FcγR^−/−^ mouse models, Gomez et al. demonstrate that murine FcγRIII (homolog of human FcγRIIA) contributes to viral replication and airway inflammation, whereas murine FcγRIIb (homolog of human FcγRIIb) has a protective effect as was shown by a decrease in viral titers (110). *In vitro*, RSV infection has been found to increase mFcγRII and mFcγRIII expression in murine macrophage cultures which subsequently showed enhanced phagocytosis (198).

Although the clinical relevance of FcγR SNPs has been studied intensively for auto-immune diseases (199), cancer treatment (200) and various viral infections (201–204), there is no data on the role of these polymorphisms in RSV infection or disease. In a genetic association study, performed to identify genes that are involved in RSV susceptibility, a SNP in FCER1A was found (205). This polymorphism had previously been found to be associated with altered FcεRI expression levels and allergic disease, supporting the involvement of IgE in RSV-mediated disease.

## Implications for Vaccine and mAb Development

Currently there are no market-approved vaccines or antivirals available against RSV. The only available treatment is the administration of a prophylactic F protein-specific mAb (Palivizumab) to reduce hospitalization in high-risk infants (206). However, the use of Palivizumab is restricted and its cost-effectiveness is often discussed (207). Improved mAbs with higher efficacy rates are thus highly needed and many research efforts are ongoing to develop these mAbs. A recent clinical trial with a pre-F-specific mAb (Suptavamab) failed to demonstrate efficacy in pre-term infants although the mAb was superior to Palivizumab in neutralization tests *in vitro* and in reducing viral load in the cotton rat model (208) (press release Regeneron, August 14, 2017). The failure of this highly neutralizing mAb indicates that protection against RSV-mediated disease, which is known to be immunopathological in nature, depends on more than just neutralization of the virus.

In addition to efforts made to develop improved therapeutic mAbs, there is an extensive pipeline of vaccines that are currently being tested in different phases of clinical development (https://www.path.org/resources/rsv-vaccine-and-mab-snapshot/). The development of vaccines is of great importance, especially for developing countries where RSV-related mortality is high and mAb therapy is inaccessible due to high costs. The majority of vaccine candidates currently in clinical trials are designed to induce systemic IgG, mostly against the RSV F protein. The results of the pre-F-specific Suptuvamab and the recent failures of two F-specific vaccine candidates tested in elderly, imply that a broader and more polyfunctional immune response may be needed to confer protection against RSV-mediated disease (209, 210) (press release Novavax, September 15, 2016).

**Figure 9:**
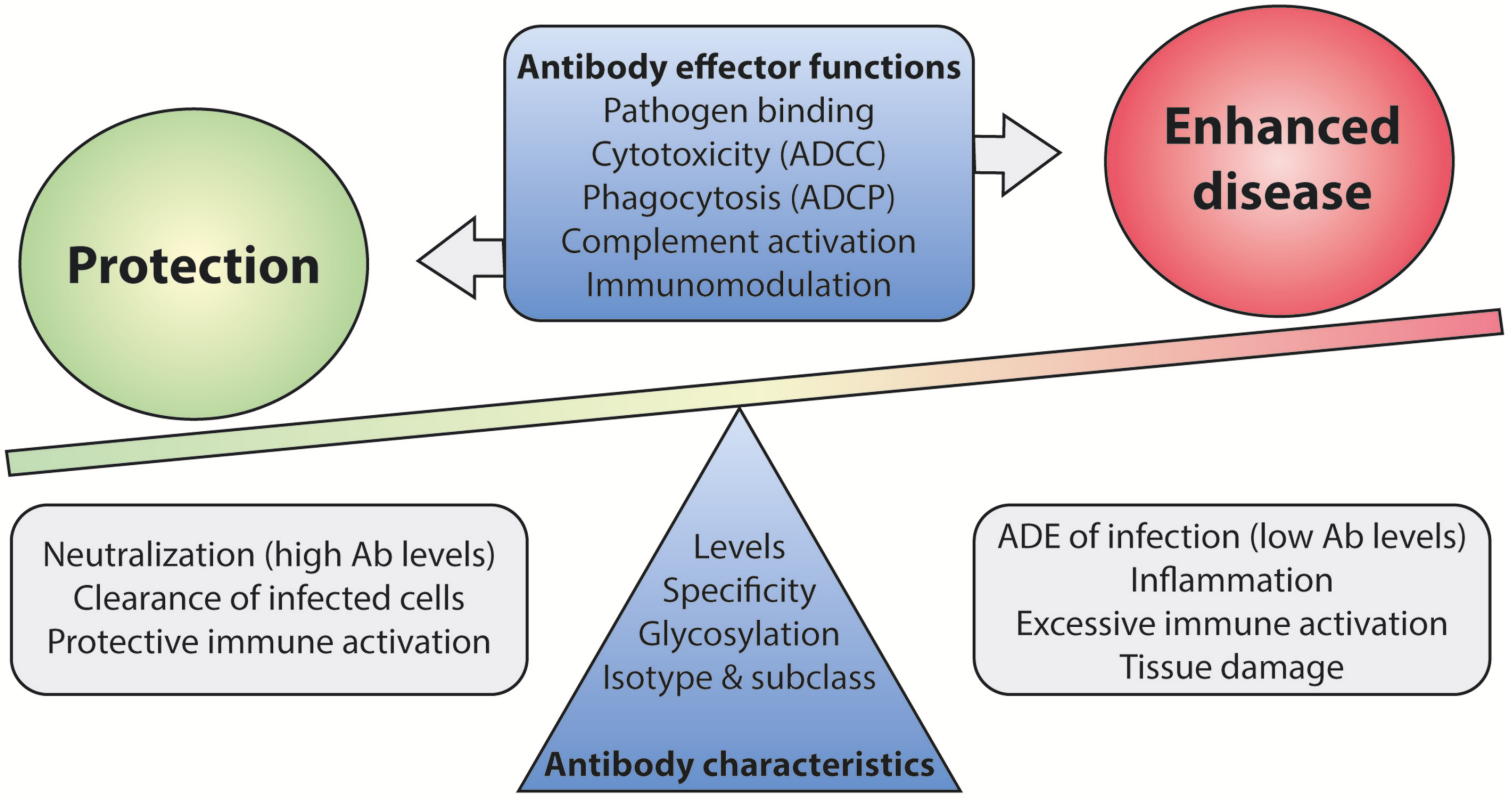
The balance between Fc-mediated protection and enhanced disease. Antibody effector functions, regulated by differences in antibody characteristics, are suspected to play a role in disease outcome upon RSV infection. Immune activation by Fc-mediated effector functions is likely needed for efficient viral clearance. However, excessive activation may lead to inflammation and tissue damage. A balanced and contained immune response is most likely the key to protection upon infection. Ab, antibody; ADCC, antibody-dependent cell-mediated cytotoxicity; ADCP, antibody-dependent cellular phagocytosis; ADE, antibody-dependent enhancement.

To this date, no accurate correlate of protection has been defined for RSV infection as virus-specific antibody levels or neutralization titers do not seem of use in this respect. The lack of a well-defined correlate of protection complicates the development of new vaccines, as efficacy now has to be demonstrated in expensive large-scale clinical trials. Mounting evidence suggests that antibody effector functions beyond neutralization can contribute to both protection and disease (110, 159, 181, 211). A balanced activation of different Fc-mediated effector functions is key to prevent excessive inflammation and tissue damage (Figure 9). It will be of importance to implement assays that identify Fc-mediated effector functions of mAbs and vaccine-induced antibodies. Studies in FcγR-knockout mice have indicated the importance of Fc-FcγR interactions for protection against RSV infection (110, 181), but the testing of mAbs and vaccines demands high-throughput approaches. Systems serology captures a wide array of antibody characteristics and effector functions. It has proven effective in identifying antibody features that contribute to protection for various (viral) pathogens (19, 212, 213). Such an approach will provide detailed information on the characteristics that are required for a protective RSV antibody response.

The ability to generate an antibody profile that selectively binds particular epitopes and FcγRs is important to enable the induction of only the desired antibody effector functions. Recent developments now allow targeted modifications to mAbs that can lead to enhancement or inhibition of specific Fc-mediated antibody effector functions through glyco-engineering or the induction of specific antibody subclasses or isotypes (159). In the future, this might also be possible for vaccines.

One can conclude from the studies presented above that Fc-FcR interactions are an integral component of the immune response against RSV and should be considered in the rational design of next generation RSV-specific mAbs and vaccines. Only limited data is available on the effect of specific Fc-mediated antibody effector functions during RSV infection, but it is clear that these can be both beneficial and detrimental for protection against RSV infection and disease outcome. In the future, Fc-mediated effector functions might be harnessed to optimize the efficacy of RSV-specific mAbs and vaccine-induced antibodies. However, our current knowledge on the precise role of individual effector functions in RSV disease is too limited to rationally design such antibodies and vaccines. Therefore, until the individual contributions of Fc-mediated effector functions to protection and disease are unraveled, aiming to induce highly neutralizing antibodies seems the safest approach. These antibodies will need to halt the infection at the site of entry and thereby prevent excessive (antibody-mediated) immune activation. It remains to be seen whether complete neutralization can be achieved via the induction of serum IgG alone, or whether the induction of mucosal IgA is necessary for reliable neutralization activity. The many clinical trials that are currently ongoing with maternal and neonatal vaccine candidates will show whether these approaches indeed result in protection during the first, most vulnerable, months of life.

## Concluding Remarks

Neutralizing antibody titers do not adequately correlate with protection against RSV disease. Interestingly, antibodies have additional Fc-mediated effector functions besides neutralization, but this area of research is currently underappreciated in the RSV field. With this review, we aim to encourage a paradigm shift from neutralization-based studies toward functional studies examining the precise role of Fc-mediated antibody effector functions in vaccine efficacy and RSV disease. We have evaluated the current literature on the effect of RSV-specific antibodies on NK cells, phagocytes, the complement system, cytokine production, and B- and T-cell skewing. Multiple *in vivo* studies using FcγR-knockout mice or modified RSV-specific antibodies indicate the importance of Fc-mediated effector functions in protection from RSV infection and disease (110, 159, 181, 211). In addition, Fc-mediated effector functions might have a role in ADE of RSV disease (22, 23). However, most studies into vaccine and mAb efficacy still only report antibody (neutralization) titers and disregard any Fc-mediated effector functions. The importance of these antibody effector functions has already been shown for multiple clinically important viral pathogens and is only starting to be explored for RSV. In our view, a better understanding of the broad range of effector mechanisms that are induced by RSV-specific antibodies will greatly contribute to the much-needed development and testing of next generation mAbs and vaccines against this virus.

## Author Contributions

EvE, WL, and PvK conceived the topic and scope of this review. EvE researched the literature and designed the figures. EvE and PvK drafted the first version of the manuscript. WL and GF critically reviewed the manuscript. All authors approved the final manuscript.

### Conflict of Interest Statement

The authors declare that the research was conducted in the absence of any commercial or financial relationships that could be construed as a potential conflict of interest.
